# Targeting the HPGDS-PGD_2_ axis to treat Th17 cell-mediated autoimmunity in multiple sclerosis

**DOI:** 10.1038/s41392-026-02937-5

**Published:** 2026-10-01

**Authors:** Yuxin Zhuang, Kawai Lei, Yan Li, Wende Deng, Jianmin Yue, Jianbo Wan, Qibiao Wu, Qianjin Lu, Yun Tang, John E. J. Rasko, Juan Liu, Olivia Monteiro, Xiqing Bian, Wei Qiu, Ting Li

**Affiliations:** 1 State Key Laboratory of Mechanism and Quality of Chinese Medicine/Macau Institute for Applied Research in Medicine and Health, School of Pharmacy, Faculty of Medicine, Macau University of Science and Technology, Macau, SAR China; 2 Macau University of Science and Technology Zhuhai MUST Science and Technology Research Institute, Zhuhai, China; 3 Faculty of Chinese Medicine, Macau University of Science and Technology, Macau, SAR China; 4 The Second Affiliated Hospital of Guizhou University of Traditional Chinese Medicine, Guiyang, China; 5 State Key Laboratory of Drug Research, Shanghai Institute of Materia Medica, Chinese Academy of Sciences, Shanghai, China; 6 State Key Laboratory of Quality Research in Chinese Medicine, Institute of Chinese Medical Sciences, University of Macau, Taipa, Macau, SAR China; 7 Hospital for Skin Diseases, Institute of Dermatology, Chinese Academy of Medical Sciences and Peking Union Medical College, Nanjing, China; 8 Shanghai Key Laboratory of New Drug Design, School of Pharmacy, East China University of Science and Technology, Shanghai, China; 9 Biomedical Pioneering Innovation Center (BIOPIC), and School of Life Sciences, Peking University, Beijing, China; 10 Artificial Intelligence Cross Disciplinary Research Institute, Macau University of Science and Technology, Macau, China; 11 Faculty of Medicine, Macau University of Science and Technology, Macau, SAR China; 12 Department of Neurology, Third Affiliated Hospital of Sun Yat-Sen University, Guangzhou, China

**Keywords:** Target validation, Lymphocytes

## Abstract

Th17 cells are central drivers of autoimmune pathology, including multiple sclerosis (MS), yet the endogenous metabolic signals that shape their pathogenic differentiation remain incompletely defined. Prostaglandin D₂ (PGD_2_) is traditionally viewed as an extracellular mediator in allergic diseases. Here, we identify PGD_2_ as a cell-intrinsic antagonist of the nuclear receptor PPAR*γ*, thereby promoting Th17 cell differentiation and exacerbating EAE pathology. PGD_2_ is selectively elevated in patients with MS compared with healthy individuals. Within the MS cohort, PGD_2_ correlates with Th17 cell frequency and disease severity. Consistent with this association, administration of PGD_2_ increases the severity of experimental autoimmune encephalomyelitis (EAE) in mice. CD4^+^ T cell-specific deletion of *Hpgds*, the key synthase for PGD_2_, impairs Th17 cell polarization and protects against EAE, whereas exogenous PGD_2_ restores both Th17 cell responses and disease susceptibility. Importantly, pharmacological inhibition of HPGDS alleviates EAE symptoms in mice. Consistently, HPGDS inhibition suppresses Th17 cell differentiation in both murine and human CD4^+^ T cells, highlighting its translational potential. Mechanistically, PGD_2_ binds directly to histidine 266 and serine 342 within the PPAR*γ* ligand-binding domain and suppresses its transcriptional activity, as demonstrated by site-directed mutagenesis and structure-function rescue assays in *Pparg*^*-/-*^ T cells. Collectively, our findings define a crucial role for the HPGDS-PGD_2_ axis in Th17 cell-mediated autoimmunity, establishing HPGDS as a promising therapeutic target for Th17 cell-mediated autoimmune diseases such as MS.

**Figure:**
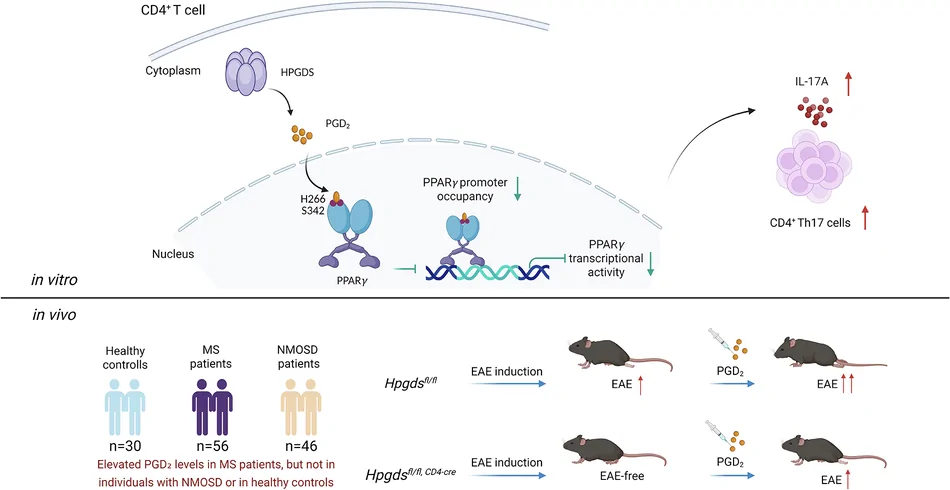

## Introduction

Th17 cells, a subset of interleukin-17 (IL-17)-secreting CD4^+^ T cells, are central drivers of autoimmune inflammation in diseases such as multiple sclerosis (MS), rheumatoid arthritis, and psoriasis.^1–4^ Among these Th17-associated autoimmune disorders, MS is the most prevalent chronic inflammatory demyelinating disease of the central nervous system (CNS), affecting millions of people worldwide. There is currently no cure for MS and the etiology remains unknown.^5,6^ In MS, Th17 cells infiltrate the CNS and mediate demyelinating injury.^7^ Consistently, the pathogenic role of Th17 cells in MS has been further substantiated by observations that IL-17-producing cells are abundant in active MS lesions.^7,8^

Since the initial discovery, Th17 cells have been distinguished from classical Th1 and Th2 cell lineages by the master transcription factor ROR*γ*t and the production of IL-17A.^9,10^ Th17 cell differentiation is driven by cytokines including IL-6, TGF-*β*, and IL-23, which establish the pathogenic transcriptional program.^11,12^ Beyond cytokine signaling, metabolic programs have emerged as key regulators of T cell lineage commitment and effector function.^13,14^ This paradigm shift has shown that metabolic pathways are not merely supportive during T cell activation. Instead, they actively shape lineage decisions by producing specific metabolites that regulate signaling cascades and epigenetic landscapes. Moreover, distinct metabolite-sensing pathways cooperate dynamically with lineage-defining transcription factors to establish and stabilize cell identity.^13,15^ Through this metabolic-transcriptional integration, metabolite availability is transduced into durable epigenetic signatures that govern functional plasticity and commitment, underscoring that cellular metabolism is an active determinant rather than a passive supporter of adaptive immune responses.^13,16–18^ For instance, de novo fatty acid synthesis, mediated by ACC1, is required for Th17 cell polarization.^19^ Notably, short-chain fatty acids (SCFAs) potentiate Th17 cell responses, whereas 3-oxoLCA inhibits their differentiation.^20^ Similarly, long-chain fatty acid oxidation preferentially supports the survival and suppressive function of regulatory T cells and memory T cells by fueling mitochondrial respiration.^14^ These findings highlight the importance of immunometabolism in shaping autoimmune pathology, yet the contribution of endogenous lipid metabolites to Th17-driven human diseases such as MS remains incompletely understood.

As a classical pathway of lipid metabolism, arachidonic acid (AA) signaling generates a range of bioactive lipid mediators, such as prostaglandins, leukotrienes, and hydroxy eicosatetraenoic acids (HETEs), which are potent immunomodulators with well-established roles in innate and stromal cells. However, their roles within T cells, particularly as intracellular regulators of lineage fate, are poorly characterized. Prostaglandin D₂ (PGD_2_) is classically recognized as an extracellular lipid produced by mast cells that signals via surface receptors such as prostaglandin D2 receptor 2 (CRTH2) to promote allergic inflammation.^21,22^ Whether PGD_2_ can be endogenously synthesized by T cells and act intracellularly to reprogram transcription has not been explored previously. Peroxisome proliferator-activated receptor gamma (PPAR*γ*) plays a key role in lipid metabolism, and synthetic PPAR*γ* agonists have shown therapeutic potential in inflammatory diseases.^23,24^ However, how endogenous lipid metabolites engage PPAR*γ* within T cells to shape pathogenic Th17 cell responses in human autoimmune disease remains unknown.

In this study, we identify a T cell intrinsic lipid-nuclear receptor axis in which PGD_2_ synthesized via hematopoietic prostaglandin D synthase (HPGDS) directly antagonizes PPAR*γ* to promote Th17 cell differentiation and aggravate autoimmunity. Using a multi-temporal experimental strategy, we demonstrate that the HPGDS-PGD_2_ axis contributes to Th17-driven pathology throughout the disease continuum, from initial T cell priming to sustained neuroinflammation. Pharmacologic HPGDS inhibition alleviates EAE and limits Th17 cell differentiation in human and mouse CD4^+^ T cells, highlighting its translational potential. Together, these findings reveal a previously unrecognized mechanism through which intracellular lipid metabolites regulate nuclear receptor activity to shape adaptive immunity, and establish the HPGDS-PGD_2_ axis as a clinically relevant and druggable pathway for Th17-mediated autoimmune diseases such as MS.

## Results

### Elevated PGD_2_ in plasma of MS patients is associated with Th17 cell frequency and disease severity

**Fig. 1:**
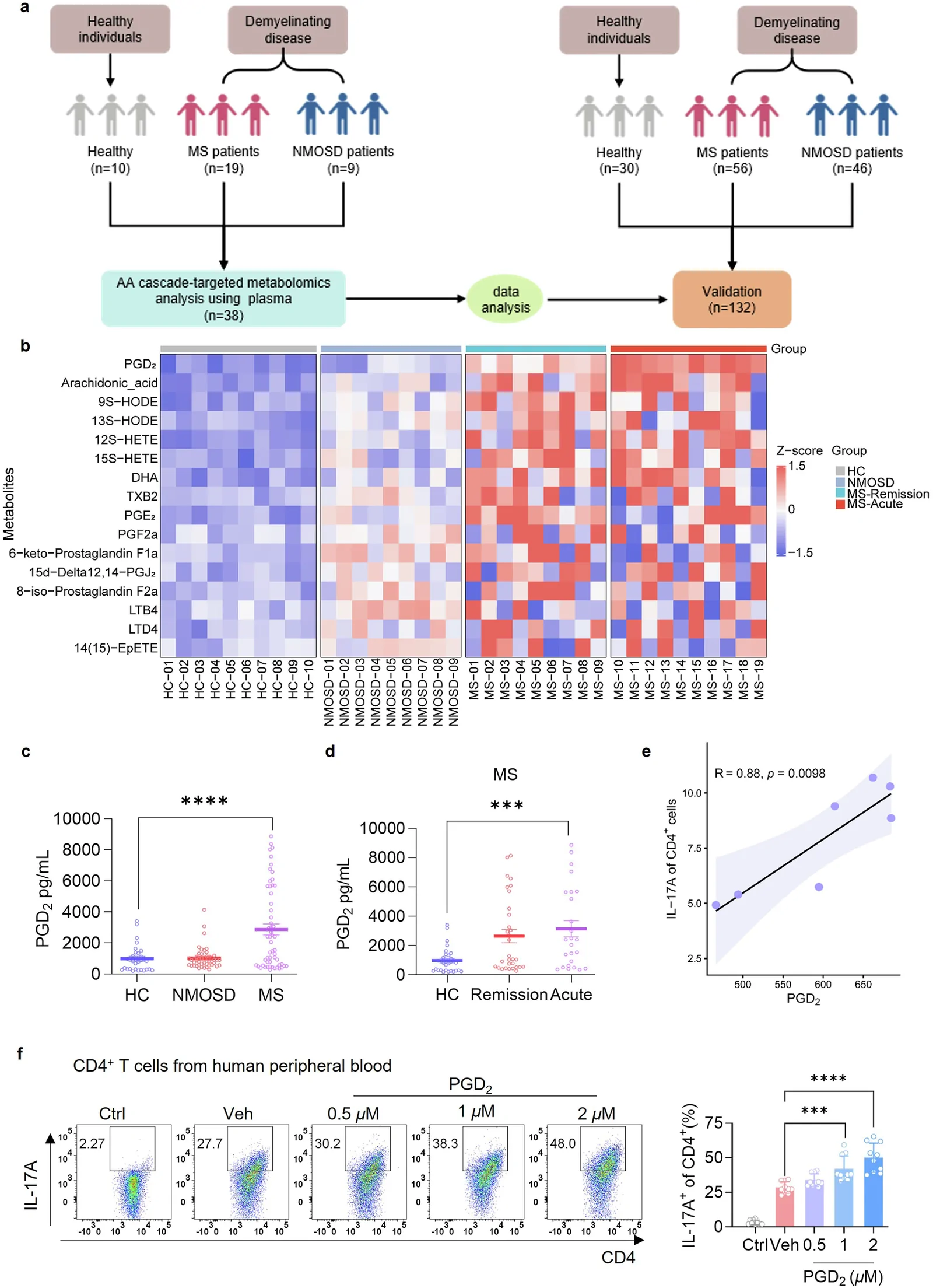
PGD_2_ selectively accumulates in MS patient plasma and correlates with Th17 cell frequency and disease severity. **a** Study design showing patient cohorts and plasma sample usage for metabolomics and ELISA. **b** Targeted eicosanoid profiling of plasma from acute MS (*n* = 10), remission MS (*n* = 9), NMOSD (*n* = 9), and healthy donors (*n* = 10). Data are represented as mean ± SEM. **c**, **d** PGD_2_ concentrations in plasma from MS (*n* = 56), NMOSD (*n* = 46), and healthy donors (*n* = 30), measured by ELISA. Data are represented as mean ± SEM. ***p <0.001, ****p < 0.0001. **e** Correlation between plasma PGD_2_ levels and Th17 cell frequency in MS patients. **f** CD4^+^ T cells isolated from MS patients were cultured under Th17 cell-polarizing conditions with increasing PGD_2_ concentrations for 72 h. Frequencies of IL-17A⁺ CD4^+^ T cells were assessed by flow cytometry (*n* = 10, each data point represents an independent biological replicate derived from a distinct individual). ***p < 0.001, ****p < 0.0001

To investigate the role of AA-derived metabolites in MS, we performed targeted lipid metabolomics followed by ELISA validation on plasma samples from patients with MS (*n* = 56), neuromyelitis optica spectrum disorder (NMOSD, *n* = 46), and matched healthy controls (*n* = 30), recruited from two independent clinical centers (Fig. 1a, Supplementary Tables 1–4). NMOSD is an inflammatory demyelinating disease that shares clinical manifestations with MS but has a distinct pathogenesis.^25^

LC-MS-based metabolomic analysis demonstrated broad metabolic alterations in patients with MS compared with NMOSD patients and healthy controls, with 10 of the 16 detected metabolites significantly elevated in the plasma of MS patients (Fig. 1b). Among these metabolites, plasma PGD_2_ was the most prominently increased in MS patients compared with NMOSD and healthy controls. This elevation was validated by ELISA assay (Fig. 1c). Multivariable logistic regression confirmed that higher PGD_2_ remained significantly associated with MS after adjustment for age, sex, current clinical status, medication use, and disease duration (Supplementary Fig. 1). Sensitivity analyses restricted to RRMS patients only (*n* = 93), restricted to patients in remission (*n* = 62), or excluding glucocorticoid users (*n* = 69) confirmed that plasma PGD_2_ remained significantly associated with MS diagnosis (Supplementary Fig. 2). Notably, within the MS cohort, PGD_2_ levels were significantly higher in the acute phase than in the remission phase (Fig. 1d). Pearson correlation analysis confirmed that PGD_2_ levels were significantly associated with disease status (acute vs. remission) within the MS cohort (Table 1). Partial correlation analysis adjusting for age, sex, and medication use confirmed that the association between plasma PGD_2_ and Expanded Disability Status Scale (EDSS) remained statistically significant (Supplementary Fig. 3).

**Table 1:** Pearson correlation analysis between metabolites abundance and disease status (acute vs. remission) in MS patients

Metabolites	Disease status (acute vs. remission)		
	*p* value^a^	95% CI^b^	*r*
Arachidonic acid	0.5391	–0.3262 to 0.5659	0.1503
9S-HODE	0.1029	–0.7147 to 0.08302	-0.3857
13S-HODE	0.9308	–0.4371 to 0.4710	0.0214
12S-HETE	0.8331	–0.4121 to 0.4944	0.0518
15S-HETE	0.6666	–0.3661 to 0.5343	0.1057
PGD_2_	<0.0001	0.6097 to 0.9339	0.8332
DHA	0.7196	–0.5215 to 0.3813	-0.0882
TXB_2_	0.7281	–0.5195 to 0.3837	-0.0854
PGE_2_	0.1313	–0.6991 to 0.1139	-0.3589
PGF2a	0.4478	–0.5898 to 0.2937	-0.1852
6-keto-Prostaglandin F1a	0.1895	–0.6727 to 0.1628	-0.3147
15d-Δ^12,14^-PGJ_2_	0.7094	–0.3784 to 0.5240	0.0915
8-iso-Prostaglandin F2a	0.5408	–0.5654 to 0.3267	-0.1497
LTB_4_	0.7233	–0.5206 to 0.3823	-0.0870
LTD_4_	0.9908	–0.4564 to 0.4520	-0.0028
14(15)-EpETE	0.8665	–0.4864 to 0.4208	-0.0414

^a^Statistically significant after Pearson correction

^b^*CI* confidence interval

The CD4^+^ T cells were isolated from MS patients’ blood to further evaluate the functional relevance. We found that PGD_2_ concentrations positively correlated with the frequency of circulating Th17 cells of MS patients (Fig. 1e, Supplementary Fig. 4). Whereas the analysis of circulating Th17 cells reflects the peripheral immune state at the time of sampling, the ex vivo assay was used to assess the responsiveness of patient-derived CD4^+^ T cells under defined experimental conditions. In this setting, PGD_2_ selectively and dose-dependently enhanced Th17 cell polarization, rather than Th1 or Treg cell (Fig. 1f, Supplementary Fig. 5), and the clinical characteristics of this analyzed patient subset are summarized in Supplementary Table 5. These findings indicate a key role for PGD_2_ in human Th17 cell-driven autoimmunity.

### PGD_2_ exacerbates EAE via promoting Th17 cell differentiation

**Fig. 2:**
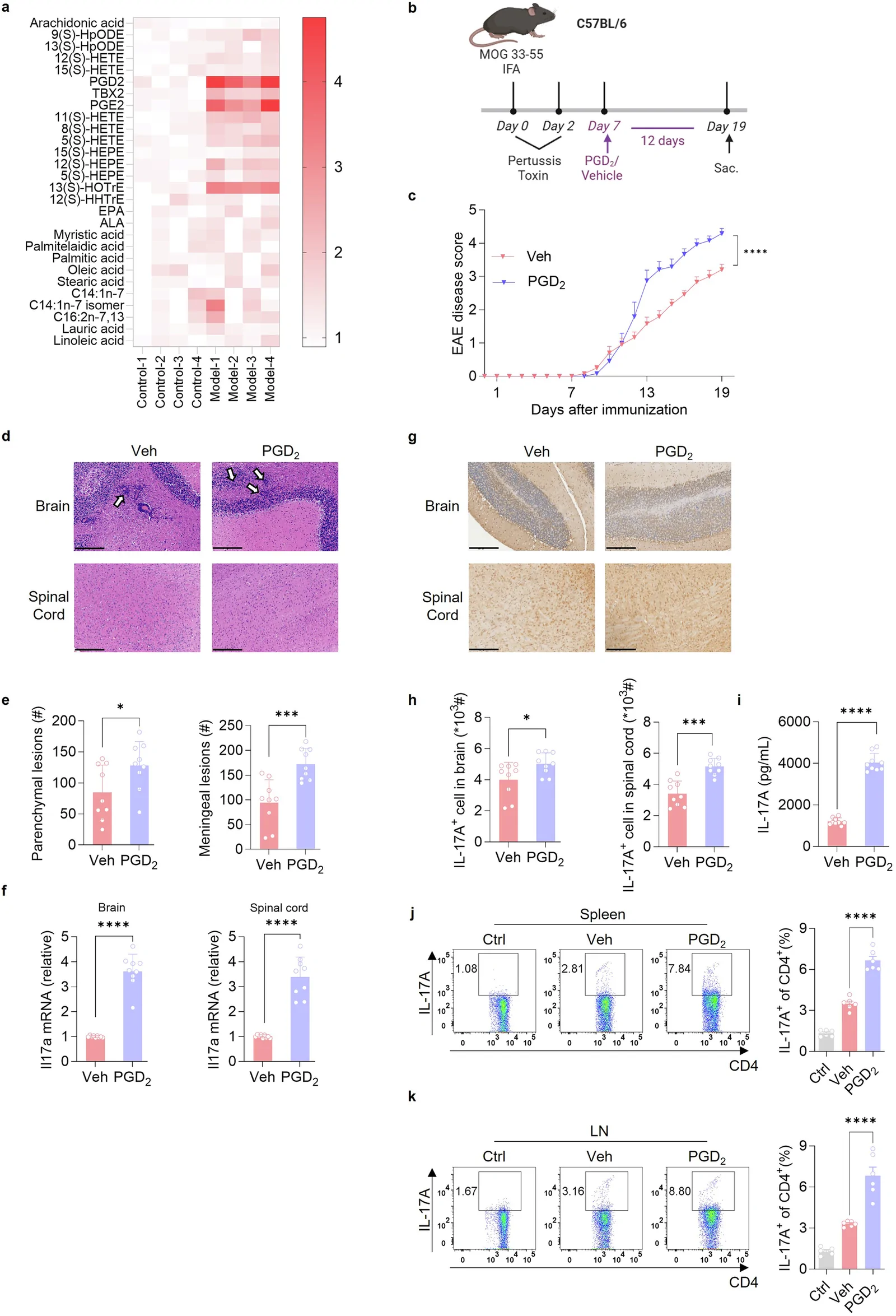
Elevated PGD_2_ levels correlate with EAE progression and promote Th17 cell differentiation. **a** Targeted eicosanoid metabolomics profiling in plasma of EAE mice at 28 days post immunization. **b** Experimental timeline for EAE induction and PGD_2_ treatment. **c** EAE clinical scores in C57BL/6 mice treated intraperitoneally with PGD_2_ (500 ng/kg) at day 7 post-immunization. (*n* = 6, two independent experiments). Data are represented as mean ± SEM. ****p < 0.0001. **d** Representative H&E-stained sections of cerebellum and spinal cord on day 19 post-immunization (×20 objective, Scale bars, 200 *μ*m). **e** Quantification of inflammatory lesions in the parenchyma and meninges of the brain and spinal cord (*n* = 9). Data are represented as mean ± SEM. *p < 0.05, ***p < 0.001. **f** *Il17a* mRNA expression in the brain and spinal cord on day 19 post-immunization (*n* = 3; three independent experiments). Data are represented as mean ± SEM. ****p < 0.0001. **g**, **h** Representative images and quantification of IL-17A immunohistochemistry in the cerebellum and spinal cord (*n* = 9) on day 19 post-immunization (×20 objective, Scale bars, 200 *μ*m). Data are represented as mean ± SEM. *p < 0.05, ***p < 0.001. **i** Plasma IL-17A concentrations measured by ELISA on day 19 post-immunization (*n* = 9). Data are represented as mean ± SEM. ****p < 0.0001. **j**, **k** Frequency of Th17 cells among CD4^+^ T cells in spleen and lymph nodes assessed by flow cytometry on day 19 post-immunization (*n* = 6). Data are represented as mean ± SEM. ****p < 0.0001

To further investigate the role of PGD_2_ in Th17 cell-mediated disease, we performed targeted metabolomics profiling in experimental autoimmune encephalomyelitis (EAE), a well-established and widely used animal model of multiple sclerosis. Among the elevated plasma metabolites in EAE mice, PGD_2_ showed the most pronounced increase (Fig. 2a), prompting us to investigate its role in Th17 cell-mediated autoimmunity.

To define optimal conditions for mechanistic studies, we first determined the dose- and timing-dependent effects of PGD_2_ administration. High-dose or early treatment markedly aggravated EAE, inducing rapid weight loss and early mortality (Supplementary Fig. 6a–d). To balance disease aggravation with experimental feasibility, we selected a 500 ng/kg dose initiated on day 7 post-immunization for subsequent experiments (Fig. 2b), because this regimen consistently and reproducibly worsened EAE severity, as reflected by clinical scores (Fig. 2c). Histological analysis revealed increased infiltration of inflammatory cells in PGD_2_-treated mice, particularly in myelinated regions of the brain and spinal cord, compared to vehicle-treated controls (Fig. 2d, e). This was accompanied by increased *Il17a* mRNA and elevated IL-17A protein levels in the brain, spinal cord and serum (Fig. 2f–i, Supplementary Fig. 7). Flow cytometric analysis showed a selective expansion of Th17 cells, but not Th1 or Treg cell populations, in the spleen of PGD_2_-treated animals (Fig. 2j, Supplementary Fig. 8a). Given the critical role of lymph nodes in T cell priming and differentiation, we also examined Th17 cell frequencies in these tissues and found similar patterns (Fig. 2k, Supplementary Fig. 8b). These results demonstrate that PGD_2_ selectively enhances Th17 cell differentiation and exacerbates neuroinflammatory pathology in vivo.

### HPGDS-derived PGD_2_ in CD4^+^ T cells drives Th17 cell differentiation

**Fig. 3:**
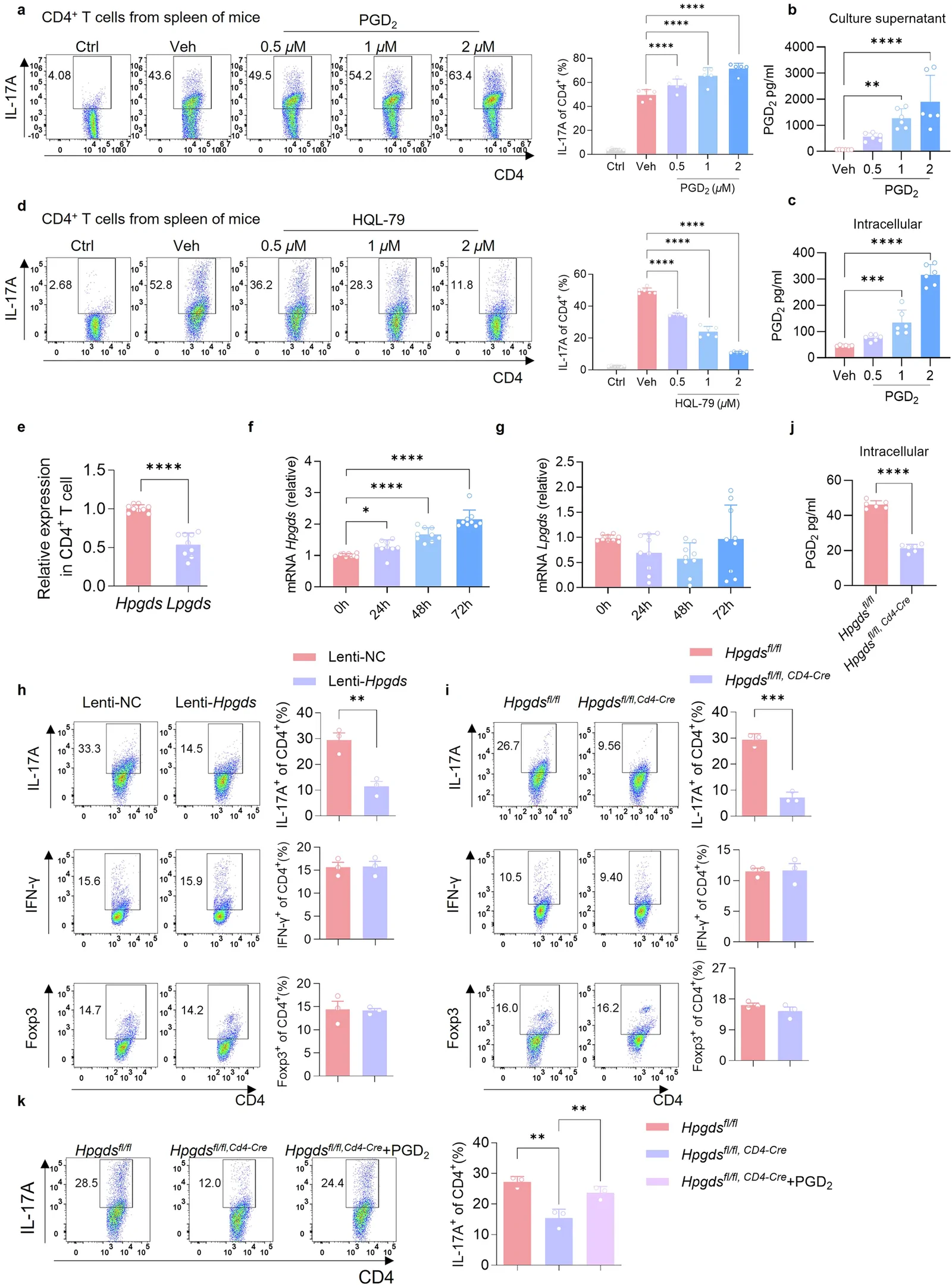
HPGDS-derived PGD_2_ in CD4^+^ cells promotes Th17 cell differentiation. **a** Naive CD4^+^ T cells from C57BL/6 spleens were cultured under Th17 cell-polarizing conditions with increasing PGD_2_ concentrations for 72 h. Frequencies of IL-17A⁺ CD4^+^ T cells were assessed by flow cytometry. (*n* = 5). Control and vehicle conditions are defined in the Methods section. Data are represented as mean ± SEM. ****p < 0.0001. **b**, **c** PGD_2_ concentrations in culture supernatants and lysates of CD4^+^ T cells cultured with increasing concentrations of exogenous PGD_2_ under Th17 cell conditions, measured by ELISA (*n* = 6). Control and vehicle conditions are defined in the Methods section. Data are represented as mean ± SEM. **p < 0.01, ****p < 0.0001. **d** Th17 cell differentiation of naive CD4^+^ T cells were cultured under Th17-polarizing conditions with increasing concentrations of the HPGDS inhibitor HQL-79 for 72 h. Frequencies of IL-17A⁺ CD4^+^ T cells were assessed by flow cytometry. (*n* = 6). Control and vehicle conditions are defined in the Methods section. Data are represented as mean ± SEM. ****p < 0.0001. **e** *Hpgds* and *Lpgds* mRNA expression in CD4^+^ T cells from spleens of C57BL/6 mice (*n* = 3; three independent experiments). Data are represented as mean ± SEM. ****p < 0.0001. **f**, **g** Time-course analysis of *Hpgds* and *Lpgds* expression in naive CD4^+^ T cells under Th17 cell-polarizing conditions (*n* = 3; three independent experiments). Data are represented as mean ± SEM. *p < 0.05, ****p < 0.0001. **h** Effect of *Hpgds* knockdown on Th17, Th1, and Treg cells differentiation (*n* = 3). Data are represented as mean ± SEM. **p < 0.01. **i** Naive CD4^+^ T cells from *Hpgds*^*fl/fl;Cd4-Cre*^ and control *Hpgds*^*fl/fl*^ mice were cultured under Th17, Th1, or Treg cells conditions. The frequencies of each subset were assessed by flow cytometry (*n* = 3). Data are represented as mean ± SEM. ***p < 0.001. **j** Intracellular PGD_2_ levels under Th17 cell-polarizing conditions were measured by ELISA (*n* = 6). Data are represented as mean ± SEM. ****p < 0.0001. **k** Exogenous PGD_2_ partially rescues Th17 cell differentiation in *Hpgds*-deficient CD4^+^ T cells. Naïve CD4^+^ T cells from *Hpgds*^*fl/fl;Cd4-Cre*^ and control *Hpgds*^*fl/fl;*^ mice were cultured under Th17-polarizing conditions for 72 hours with or without exogenous PGD_2_ (2 *μ*M). Frequencies of IL-17A⁺ CD4^+^ T cells were assessed by flow cytometry (*n* = 3). Data are represented as mean ± SEM. **p < 0.01

To explore the role of PGD_2_ in T cell function, we cultured mice naïve CD4^+^ T cells under defined polarizing conditions with increasing concentrations of exogenous PGD_2_. PGD_2_ dose-dependently and selectively increased the differentiation of Th17 cells, without significantly affecting Th1 or Treg cells (Fig. 3a, Supplementary Fig. 9). Intracellular PGD_2_ exhibited a clear dose-dependent increase that correlated with enhanced Th17 differentiation (Fig. 3b, c), with approximately 10% of exogenous PGD_2_ internalized, indicating a potential intracellular mechanism of action.

PGD_2_ can be enzymatically generated by either HPGDS or lipocalin-type PGDS (LPGDS).^26^ To distinguish their contributions, we treated cells with the selective HPGDS inhibitor HQL-79 or the LPGDS inhibitor AT-56.^27,28^ HPGDS inhibition significantly reduced Th17 cell differentiation, whereas LPGDS inhibition had no significant effect (Fig. 3d, Supplementary Fig. 10). Consistently, *Hpgds* mRNA was more highly expressed than *Lpgds* in CD4^+^ T cells (Fig. 3e), and expression of *Hpgds*, but not *Lpgds*, positively correlated with Th17 cell differentiation across conditions (Fig. 3f, g, Supplementary Fig. 11). These results imply that HPGDS is the predominant source of PGD_2_ driving Th17 cell differentiation in CD4^+^ T cells.

We further confirmed these findings by knocking down *Hpgds* in vitro, which selectively impaired Th17 cells but not Th1 or Treg cell differentiation (Fig. 3h), despite HPGDS being detectable across Th1, Treg, and Th17 cell subsets (Supplementary Fig. 12). CD4^+^ T cells from *Hpgds*^*fl/fl;Cd4-Cre*^ mice also exhibited significantly reduced Th17 cell differentiation under Th17 cell-polarizing conditions, with no effect on other subsets (Fig. 3i). In line with the impaired Th17 cell response, PGD_2_ levels were markedly decreased in *Hpgds*-deficient CD4^+^ T cells compared to *Hpgds*^*fl/fl*^ counterparts (Fig. 3j), confirming that HPGDS is essential for PGD_2_ production in CD4^+^ T cells. Exogenous PGD_2_ partially rescued Th17 cell differentiation in *Hpgds*-deficient CD4^+^ T cells (Fig. 3k), indicating that loss of PGD_2_ is a major contributor to the impaired Th17 cell differentiation caused by *Hpgds* deficiency.

Together, these results demonstrate that CD4^+^ T cell-intrinsic HPGDS promotes Th17 cell differentiation by serving as the major source of PGD_2_.

### Conditional deletion of *Hpgds* in CD4^+^ T cells protects against EAE by suppressing Th17 cell responses

**Fig. 4:**
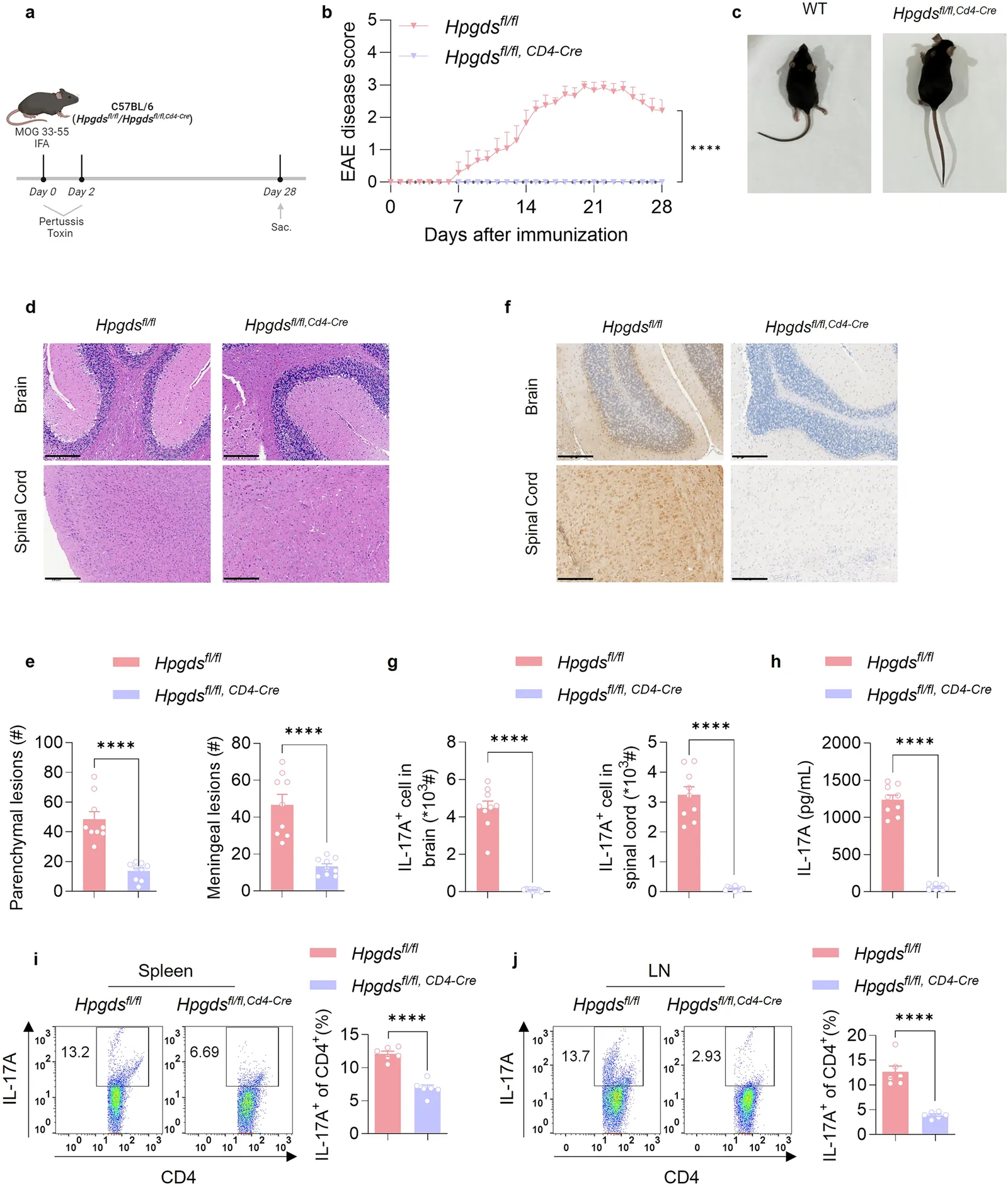
CD4^+^ T cell-specific deletion of *Hpgds* inhibits Th17 cell differentiation and protects against EAE. **a** Experimental timeline for EAE induction in *Hpgds*^*fl/fl;Cd4-Cre*^ and control mice. **b** EAE clinical scores in both genotypes (*n* = 6, two independent experiments). Data are represented as mean ± SEM. ****p < 0.0001. **c** Representative clinical appearance of mice on day 28 post-immunization. **d** Representative H&E-stained sections of the cerebellum and spinal cord (×20 objective, Scale bars, 200 *μ*m). **e** Quantification of inflammatory lesions in the parenchyma and meninges of the brain and spinal cord (*n* = 9). Data are represented as mean ± SEM. ****p < 0.0001. **f**, **g** IL-17A immunohistochemistry and quantification in the cerebellum and spinal cord (×20 objective, Scale bars, 200 *μ*m, *n* = 9). Data are represented as mean ± SEM. ****p < 0.0001. **h** Plasma IL-17A concentrations measured by ELISA on day 28 post-immunization (*n* = 3, three independent experiments). Data are represented as mean ± SEM. ****p < 0.0001. **i**–**j** Frequencies of Th17 cell in spleen and lymph nodes analyzed by flow cytometry (*n* = 6). Data are represented as mean ± SEM. ****p < 0.0001

To assess the in vivo relevance of CD4^+^ T cell-intrinsic HPGDS, we examined its role in EAE and Th17 cell polarization. Unlike the exogenous PGD_2_ supplementation experiments, in which certain dosing regimens led to excessively severe disease and required early termination, genetic *Hpgds* deletion permitted evaluation throughout the full disease course. Indeed, *Hpgds*^*fl/fl;Cd4-Cre*^ mice were almost completely protected from EAE, exhibiting minimal to no clinical signs throughout the disease course and significantly reduced inflammatory lesions in the brain and spinal cord (Fig. 4a–e, Supplementary Video 1). Consistent with these findings, the production of IL-17A in these tissues was markedly reduced (Fig. 4f, g), and IL-17A secretion was significantly decreased in the serum of *Hpgds*^*fl/fl;Cd4-Cre*^ mice, compared to their *Hpgds*^*fl/fl*^ counterparts (Fig. 4h). Flow cytometric analysis showed reduced Th17 cell frequencies in both spleen and lymph nodes, with Th1 and Treg cells unaffected (Fig. 4i–j, Supplementary Fig. 13). These observations indicate that CD4^+^ T cell-intrinsic HPGDS expression is required for Th17 cell-driven EAE pathogenesis.

### Exogenous PGD_2_ restores EAE susceptibility in *Hpgds*^*fl/fl;Cd4-Cre*^ mice by rescuing Th17 cell responses

**Fig. 5:**
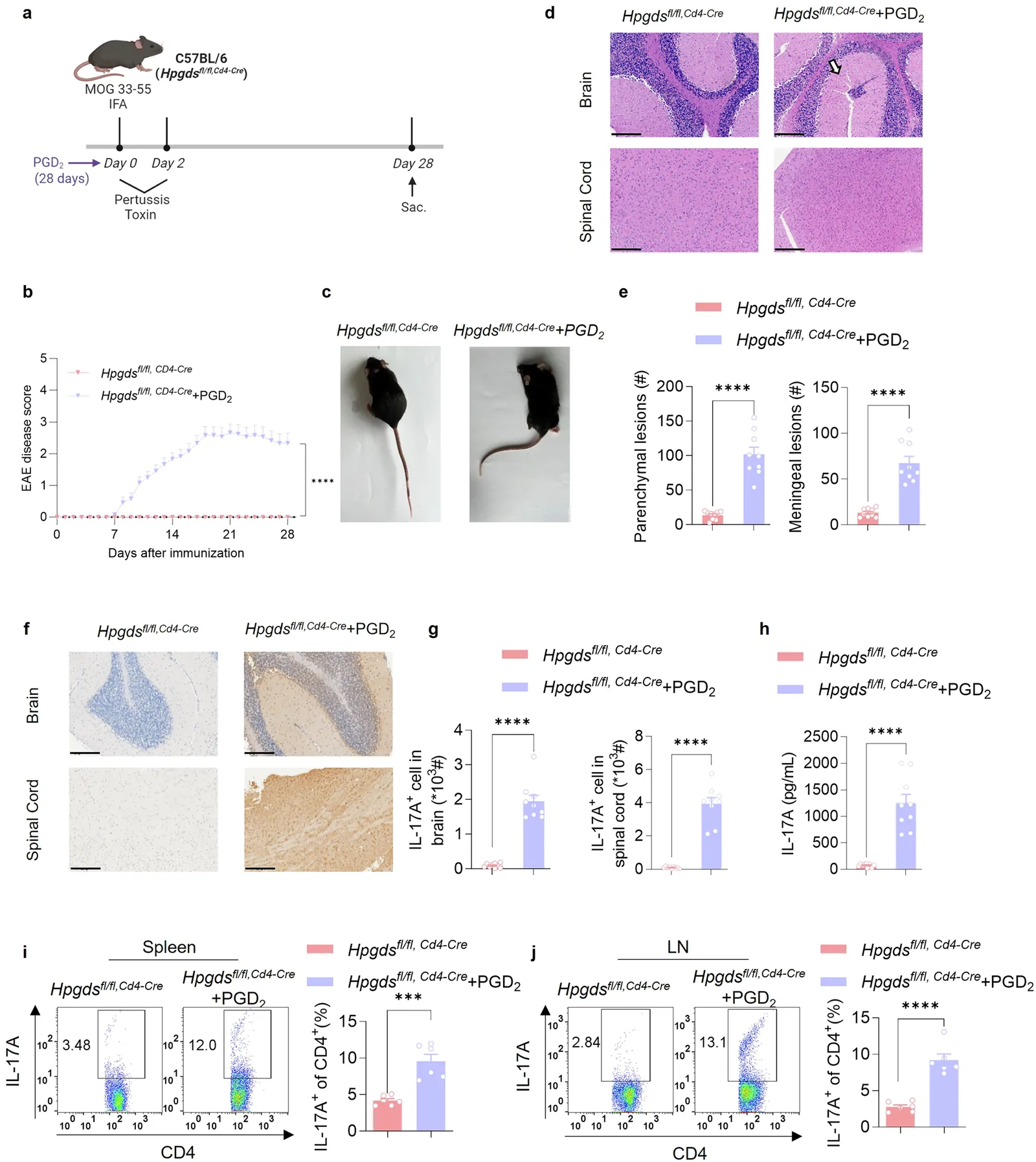
Exogenous PGD_2_ restores Th17 cell responses and EAE susceptibility in *Hpgds*^*fl/fl;Cd4-Cre*^ mice. **a** Experimental design for PGD_2_ rescue in *Hpgds*^*fl/fl;Cd4-Cre*^ mice. **b** EAE clinical scores in *Hpgds*^*fl/fl;Cd4-Cre*^ mice intraperitoneally treated with or without 1000 ng/kg PGD_2_ (*n* = 6; two independent experiments). Data are represented as mean ± SEM. ****p < 0.0001. **c** Representative clinical appearance of PGD_2_-treated and control mice. **d** H&E-stained sections of cerebellum and spinal cord on day 28 post-immunization (×20 objective, Scale bars, 200 *μ*m). **e** Quantification of inflammatory lesions in the parenchyma and meninges of the brain and spinal cord (*n* = 9). Data are represented as mean ± SEM. ****p < 0.0001. **f**, **g** IL-17A immunostaining and quantification in the cerebellum and spinal cord (×20 objective, Scale bars, 200 *μ*m, *n* = 9). Data are represented as mean ± SEM. ****p < 0.0001. **h** Plasma IL-17A concentrations assessed by ELISA (*n* = 3, three independent experiments). Data are represented as mean ± SEM. ****p < 0.0001. **i**–**j** Th17 cell frequencies in spleen and lymph nodes analyzed by flow cytometry (*n* = 6). Data are represented as mean ± SEM. ***p < 0.001, ****p < 0.0001

Given that CD4^+^ T cell-intrinsic PGD_2_ is required for Th17 cell-driven pathology, we next asked whether exogenous PGD_2_ is sufficient to compensate for its endogenous absence. In contrast to wild-type mice, *Hpgds*^*fl/fl;Cd4-Cre*^ mice lack T cell-intrinsic HPGDS-dependent PGD_2_ production, indicating a distinct endogenous PGD_2_ background in this genetic setting. Therefore, this rescue experiment was not designed to directly match the PGD_2_ supplementation regimen used in wild-type mice, but to test whether early exogenous PGD_2_ could replace the missing signal. Under the regimen used here, we administered 1000 ng/kg PGD_2_ intraperitoneally from day 0. The results demonstrated that exogenous PGD_2_ restored EAE susceptibility in *Hpgds*^*fl/fl;Cd4-Cre*^ mice (Fig. 5a–c, Supplementary Video 2), and exhibited significantly increased inflammatory infiltration in the brain and spinal cord of the mice (Fig. 5d, e), along with increased IL-17A expression (Fig. 5f, g). Consistent with these observations, IL-17A secretion and Th17 cell frequencies were increased in the *Hpgds*^*fl/fl;Cd4-Cre*^ mice treated with PGD_2_, without affecting Th1 or Treg cell frequencies (Fig. 5h–j, Supplementary Fig. 14). These results demonstrate that PGD_2_ is sufficient to restore Th17 cell responses and EAE pathology in the absence of endogenous HPGDS, underscoring the central role of the HPGDS-PGD_2_ axis in Th17 cell-mediated pathogenesis.

### PGD_2_ antagonizes PPAR*γ* via H266/S342 to promote Th17 cell polarization

To elucidate the mechanism by which PGD_2_ promotes Th17 cell differentiation, we first investigated whether it interacts with retinoic acid-related orphan receptors *γ*t (ROR*γ*t), the master transcription factor for Th17 cell lineage commitment.^29^ However, PGD_2_ failed to bind to ROR*γ*t (Supplementary Fig. 15a), and did not affect *IL17A* promoter activity in a luciferase reporter assay (Supplementary Fig. 15b), suggesting that its effect is independent of ROR*γ*t. In addition, pharmacological inhibition of the classical PGD_2_ receptors, D-prostanoid receptor 1 (DP1) and CRTH2,^30,31^ did not impact Th17 cell differentiation (Supplementary Fig. 16), suggesting that PGD_2_ promotes Th17 cell differentiation through a non-canonical, surface receptor-independent mechanism.

**Fig. 6:**
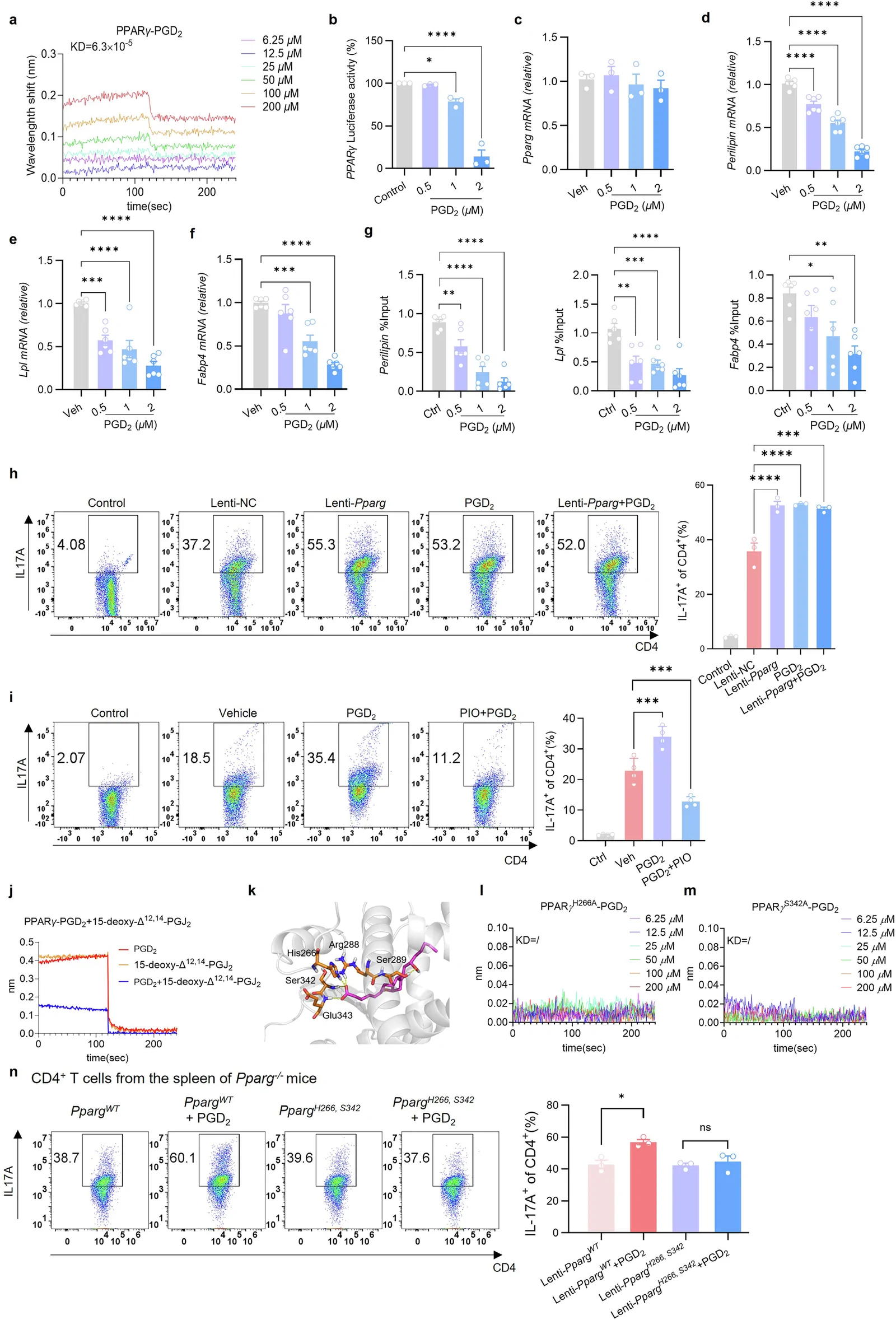
PGD_2_ antagonizes PPAR*γ* via H266 and S342 to promote Th17 cell differentiation. **a** Direct PGD_2_-PPAR*γ* interaction measured by bio-layer interferometry (BLI). **b** Luciferase activity of a PPAR*γ* reporter in response to PGD_2_ stimulation (*n* = 3). Data are represented as mean ± SEM. *p < 0.05, ****p < 0.0001. **c** Relative mRNA expression of *Pparg* in CD4^+^ T cells under Th17 cell-polarizing conditions with or without PGD_2_ (*n* = 3). Data are represented as mean ± SEM. **d**–**f** Relative mRNA expression of *Perilipin*, *Lpl*, and *Fabp4* in CD4^+^ T cells under Th17 cell-polarizing conditions with or without PGD_2_ (*n* = 3, two independent experiments). Data are represented as mean ± SEM. ***p < 0.001, ****p < 0.0001. **g** *Perlipin, Lpl and Fabp4* promoter activity in response to PGD_2_ (*n* = 6). Data are represented as mean ± SEM. *p < 0.05, **p < 0.01, ***p < 0.001, ****p < 0.0001. **h** Naive CD4^+^ T cells were transfected with lenti-*Pparg*, then cultured under Th17-polarizing conditions with or without PGD₂. Frequencies of IL-17A⁺ CD4^+^ T cells were assessed by flow cytometry. (*n* = 3). Control and vehicle conditions are defined in the “Methods” section. Data are represented as mean ± SEM. ***p < 0.001, ****p < 0.0001. **i** Th17 cell differentiation of naive CD4^+^ T cells isolated from WT mice and cultured with or without PGD_2_ (2 *μ*M) or PIO (10 *μ*M) for 72 h. Frequencies of IL-17A⁺ CD4^+^ T cells were assessed by flow cytometry. (*n* = 4). Control and vehicle conditions are defined in the Methods section. Data are represented as mean ± SEM. ***p < 0.001. **j** Competitive binding of PGD_2_ and 15d-PGJ₂ to PPAR*γ* assessed by BLI. **k** Predicted structure of the PPAR*γ*-PGD_2_ complex showing key interacting residues. **l**–**m** BLI binding curves of PGD_2_ to PPAR*γ* mutants H266A and S342A. **n** Th17 cell differentiation of *Pparg*^*–/–*^ CD4^+^ T cells reconstituted with either wild-type or H266A/S342A mutant PPAR*γ*, and treated with or without PGD_2_, assessed by flow cytometry (*n* = 3). Data are represented as mean ± SEM. *p < 0.05

Molecular docking analyses identified PPAR*γ* as a potential binding target of PGD_2_. The binding and luciferase assays confirmed that PGD_2_ directly interacted with PPAR*γ* and inhibited its transcriptional activity, without affecting *Pparg* mRNA expression in CD4^+^ T cells (Fig. 6a–c). Consistently, PGD_2_ suppressed the expression of canonical PPAR*γ* target genes, including *Perilipin*, *Lpl and Fabp4* in CD4^+^ T cells (Fig. 6d–f). ChIP-qPCR analysis further revealed that PGD_2_ reduced PPAR*γ* occupancy at the promoter regions of these target genes (Fig. 6g), supporting a direct transcriptional repression mechanism.

In line with these findings, PGD_2_ treatment failed to enhance Th17 cell differentiation in *Pparg*-deficient cells (Fig. 6h). Conversely, pioglitazone (PIO), a synthetic agonist of PPAR*γ*,^32^ blocked the PGD_2_-induced enhancement of Th17 cell polarization (Fig. 6i), further supporting the role of PGD_2_ as a PPAR*γ* antagonist.

To explore their binding interface, we performed competition binding assays, and the results showed that PGD_2_ competes with the canonical PPAR*γ* ligand 15-deoxy-Δ^12,14^-PGJ_2_ for receptor occupancy (Fig. 6j). Molecular docking predicted that PGD_2_ interacts with PPAR*γ* at a pocket defined by histidine 266 (H266), arginine 288 (R288), serine 289 (S289), serine 342 (S342), and glutamate 343 (E343) (Fig. 6k). Mutagenesis studies showed that substitution of H266 or S342 abolished PGD_2_ binding, whereas other point mutations did not affect interaction (Fig. 6l, m, Supplementary Fig. 17). To confirm binding specificity, *Pparg*^*-/-*^ CD4^+^ T cells were reconstituted with either wild-type or H266A/S342A mutant PPAR*γ*. PGD_2_ enhanced Th17 cell differentiation in wild-type-rescued cells, but this effect was abolished in cells expressing the mutant receptor (Fig. 6n). These findings support the role of PGD_2_ as a functional endogenous antagonist of PPAR*γ* that promotes Th17 cell differentiation through direct binding to H266 and S342.

### Proof-of-concept pharmacologic inhibition of HPGDS attenuates EAE and Th17 responsese

**Fig. 7:**
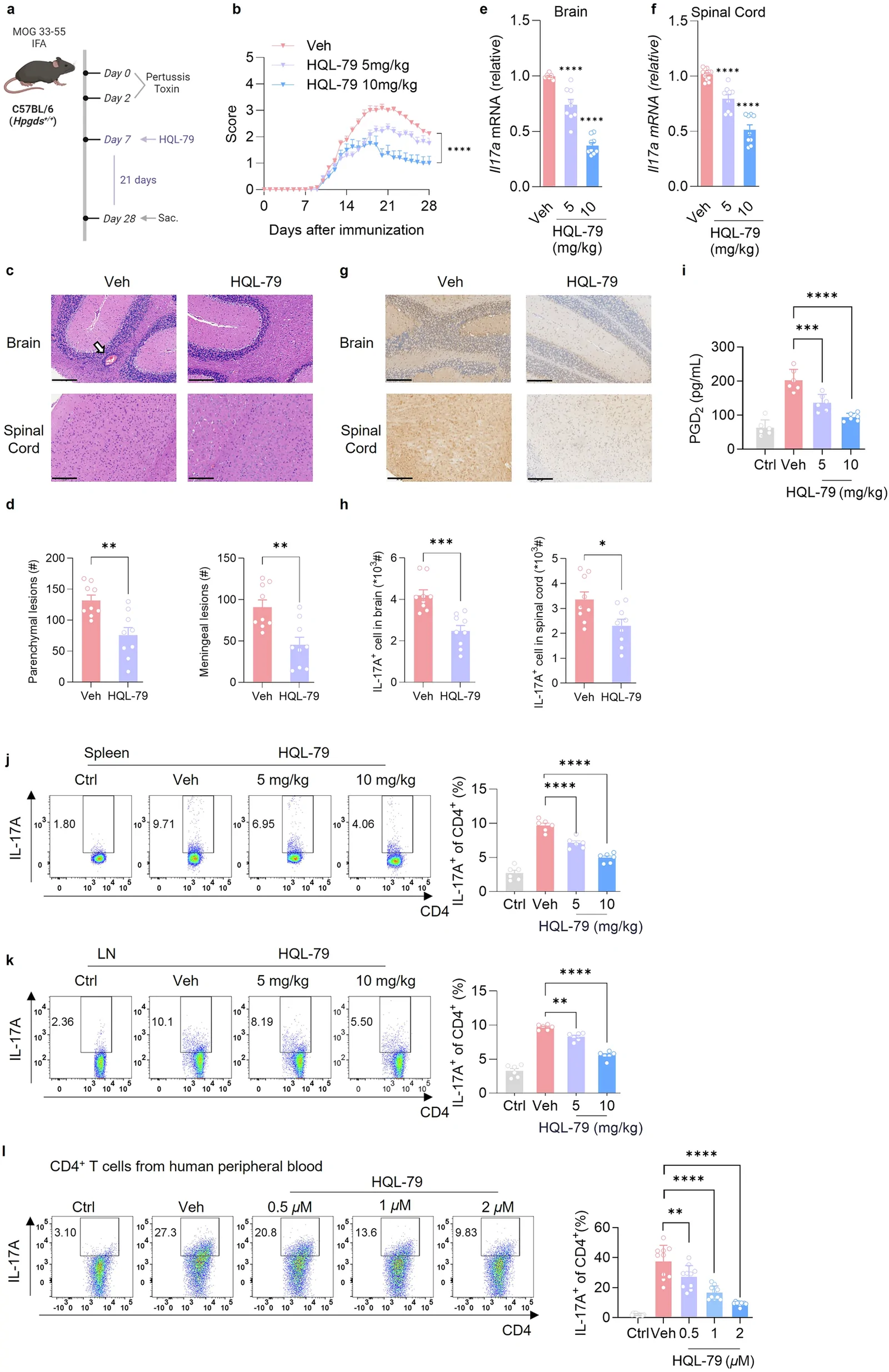
HPGDS inhibition with HQL-79 suppresses Th17 cell responses and attenuates EAE. **a** Experimental timeline for HQL-79 administration in EAE mice. **b** EAE clinical scores in C57BL/6 mice treated with HQL-79 (*n* = 6; two independent experiments). Data are represented as mean ± SEM. ****p < 0.0001. **c** Representative H&E-stained sections of cerebellum and spinal cord on day 28 (×20 objective, Scale bars, 200 *μ*m). **d** Quantification of inflammatory lesions in the parenchyma and meninges of the brain and spinal cord (*n* = 9). Data are represented as mean ± SEM. **p < 0.01. **e**, **f** *Il17a* mRNA expression in the brain and spinal cord (*n* = 3, three independent experiments). Data are represented as mean ± SEM. ****p < 0.0001. **g**, **h** IL-17A staining and quantification in the cerebellum and spinal cord (×20 objective, Scale bars, 200 *μ*m, *n* = 9). Data are represented as mean ± SEM. *p < 0.05, ***p < 0.001. **i** Plasma PGD_2_ levels in EAE mice treated with vehicle or HQL-79 (5 mg/kg or 10 mg/kg, daily from day 7) measured by ELISA on day 28 post-immunization (*n* = 6). Data are represented as mean ± SEM. ***p < 0.001, ****p < 0.0001. **j**, **k** Frequencies of Th17 cell in spleen and lymph nodes on day 28, analyzed by flow cytometry (*n* = 6). Data are represented as mean ± SEM. **p < 0.01, ****p < 0.0001. **l** CD4^+^ T cells isolated from MS patients were cultured under Th17 cell-polarizing conditions with increasing HQL-79 concentrations. Th17 cell were analyzed by flow cytometry (*n* = 10, each data point represents an independent biological replicate derived from a distinct individual). Data are represented as mean ± SEM. **p < 0.01, ****p < 0.0001

To test proof-of-concept pharmacologic inhibition of the HPGDS-PGD_2_ axis in EAE, we treated mice with HQL-79, a selective HPGDS inhibitor. We first confirmed that HQL-79 directly binds to HPGDS in vitro by biolayer interferometry (Supplementary Fig. 18). This treatment led to marked clinical improvement, along with reduced inflammatory infiltration in myelinated brain and spinal cord regions (Fig. 7a–d). Additionally, we noted a significant decrease in *Il17a* mRNA expression and IL-17A production in the brain and spinal cord following HQL-79 treatment (Fig. 7e–h). To verify target engagement in vivo, we measured plasma PGD_2_ levels in HQL-79-treated EAE mice and found that HQL-79 significantly reduced circulating PGD_2_ compared to vehicle controls (Fig. 7i). In agreement with these results, we found a significant decrease in the frequency of Th17 cells rather than Th1 or Treg cells from the spleen and LN of the mice treated with HQL-79 (Fig. 7j, k, Supplementary Fig. 19). To further evaluate the functional relevance, CD4^+^ T cells were then isolated from MS patients’ blood and treated with HQL-79. Consistent with the result in the EAE model. We found that pharmacological inhibition of HPGDS with HQL-79 suppressed Th17 cell differentiation in CD4^+^ T cells from MS patients (Fig. 7l, Supplementary Table 5). These findings suggest that the HPGDS-PGD_2_ axis actively drives Th17 cell responses in MS and may represent a viable therapeutic target in human autoimmune disease.

## Discussion

Our study uncovers a previously unrecognized lipid-nuclear receptor axis in which PGD_2_, derived from CD4^+^ T cells, contributes to Th17 cell differentiation and autoimmunity by antagonizing PPAR*γ*. This finding extends the role of PGD_2_ beyond its classical function as a CRTH2 ligand in allergic responses, revealing it as a direct intracellular modulator of nuclear receptor activity in Th17 cell-mediated autoimmunity.

Across two independent cohorts, PGD_2_ was elevated in MS and associated with disease activity and Th17-related immune features, consistent with prior observations of PGD_2_ enrichment in the cerebrospinal fluid of patients with MS.^33^ These findings raise the possibility that plasma PGD_2_, in combination with existing markers such as anti-aquaporin-4 serum antibodies (AQP4-Abs), could aid in differentiating MS from NMOSD. However, these clinical associations should be interpreted cautiously, as the human cohort data are cross-sectional and do not establish temporal or causal relationships.

We next investigated the in vivo requirement of HPGDS, the PGD_2_ synthase, in Th17 cell-mediated pathology using both genetic and pharmacologic approaches. Conditional deletion of *Hpgds* in CD4^+^ T cells conferred complete protection against EAE under experimental conditions and suppressed Th17 cell responses, while exogenous PGD_2_ restored disease susceptibility and Th17 cell differentiation. These findings identify the HPGDS-PGD_2_ axis as a key T cell-intrinsic regulator of lineage fate and position HPGDS as a tractable upstream target. Consistently, pharmacologic inhibition of HPGDS alleviated EAE and limited Th17 differentiation in human and mouse CD4^+^ T cells. In addition, HQL-79 reduced plasma PGD_2_ levels in vivo under the dosing regimen used in this study, supporting target engagement in the EAE setting. The consistent results between genetic *Hpgds* deletion and pharmacologic HPGDS inhibition support on-target specificity. However, potential off-target effects of HQL-79 cannot be fully excluded. Thus, our findings establish the HPGDS-PGD_2_ axis as a mechanistically targetable pathway, rather than providing definitive preclinical validation of HQL-79 as a therapeutic candidate.

PGD_2_ is historically linked to allergic responses, but its role in adaptive immunity remains poorly characterized, in part due to its rapid degradation in systemic circulation.^34,35^ Our study demonstrates that PGD_2_ functions as a cell-intrinsic mediator, where its local synthesis and action within CD4^+^ T cells bypass these systemic limitations. PGD_2_ signals through CRTH2 and DP1 in other immune contexts; however, its effect on Th17 differentiation in CD4^+^ T cells is independent of these surface receptors, suggesting a distinct intracellular mechanism. Our data support an autocrine/intracellular signaling model wherein PGD_2_ acts within CD4^+^ T cells to antagonize PPAR*γ*, distinct from paracrine actions via DP1/CRTH2 which do not mediate Th17 cell differentiation. Exogenous PGD_2_ partially rescued the Th17 cell defect in *Hpgds*-deficient T cells, indicating that PGD_2_ is a major mediator of the observed phenotype, although contributions from other HPGDS-dependent metabolites cannot be excluded.

Prior studies showed that *Ptgdr* deletion in dendritic cells modulates EAE,^36^ and our findings uncover a parallel, cell-intrinsic pathway in T cells, supporting compartment-specific PGD_2_ signaling in Th17-mediated autoimmunity.

Our data also demonstrate that PGD_2_ acts independently of ROR*γ*t, the master transcription factor for Th17 cell differentiation.^29^ Instead, PGD_2_ can antagonize PPAR*γ*, a nuclear receptor previously shown to inhibit Th17 cell differentiation.^32^ This is consistent with previous findings that pharmacologic PPAR*γ* agonists, such as pioglitazone, suppress Th17 cell responses and attenuate EAE severity.^37^

Due to the cross-sectional design, longitudinal clinical parameters (e.g., annual relapse rates, exacerbations before vs. during DMT) were not captured. Additionally, DMT duration was not recorded and could not be adjusted. A limitation of the genetic loss-of-function experiments is that *Hpgds*^*fl/fl;Cd4-Cre*^ mice were compared to *Hpgds*^*fl/fl*^ littermates but not to mice expressing *Cd4-Cre* alone. Studies using this additional control group would further validate that the observed phenotype is specifically attributable to *Hpgds* deletion in CD4^+^ T cells. In addition, the murine experiments were not designed as a longitudinal, phase-resolved analysis of EAE, and therefore do not permit formal assessment of PGD_2_ kinetics across the disease course or stage-specific correlation with disease severity. Prospective clinical studies and dedicated longitudinal analyses in EAE will be needed to address these limitations.

In addition, although the ex vivo human CD4^+^ T-cell experiments support the relevance of this pathway in patients with MS, these mechanistic data should still be considered exploratory and will require confirmation in larger cohorts.

The combination of acute-phase interventions and genetic deletion models demonstrates that the HPGDS-PGD_2_ axis contributes to disease, from acute MS to sustained EAE pathology. Notably, the in vivo EAE experiments were not designed as a single stage-matched model of acute MS, but as complementary mechanistic tests of sufficiency, necessity, and rescue under distinct experimental contexts. Accordingly, differences in intervention timing and study endpoint were determined by the specific mechanistic question being addressed and, where applicable, by phenotype severity and humane endpoint criteria. In the present study, we focused on EAE as a representative model of Th17-driven CNS autoimmunity that is most directly aligned with the clinical MS component of our work. Whether similar protective effects extend to other Th17-associated autoimmune or inflammatory settings remains an important question for future investigation. Given that the human clinical data are cross-sectional and associative, PGD_2_ modulation should be explored as an adjunct to existing DMTs rather than a standalone therapy. Its potential utility as a disease activity biomarker requires prospective validation with formal ROC/AUC analyses.

In summary, we define a CD4^+^ T cell-intrinsic lipid-nuclear receptor axis in which HPGDS-derived PGD_2_ antagonizes PPAR*γ* to drive Th17 cell polarization and autoimmunity. By linking this pathway to disease activity in both mice and humans and demonstrating its tractability to pharmacologic intervention, our work provides a mechanistic and translational foundation for targeting the HPGDS-PGD_2_ axis as a potential therapeutic strategy in Th17 cell-driven autoimmune diseases, such as MS.

## Materials and methods

### Study design

The objective of this study was to reveal the role of HPGDS-PGD_2_-PPAR*γ* axis as a druggable checkpoint with therapeutic potential in Th17 cell-driven neuroinflammation and characterize the therapeutic potential of HPGDS for the treatment of autoimmune diseases. We used *Hpgds*^*fl/fl, Cd4-Cre*^ mice to establish the experimental autoimmune encephalomyelitis (EAE) model. We found CD4^+^ T cell-specific deletion of *Hpgds* suppresses Th17 cell differentiation and protects against disease, whereas exogenous PGD_2_ restores both Th17 cell responses and EAE susceptibility. Pharmacological HPGDS inhibition mirrors the protective effects of genetic ablation. We used a multi-pronged strategy, including molecular docking, biolayer interferometry, mutagenesis-based structure-function rescue, luciferase reporter assays, and ChIP-qPCR, to confirm that PGD_2_ was a PPAR*γ* natural antagonist. The sample size for each experiment is specified in the figure legends. The number of independent experiments performed is stated in the figure legends.

### Antibodies and reagents

The following reagents were purchased from the indicated sources: Brefeldin A Solution (Biolegend, 420601), Foxp3/Transcription Factor Staining Buffer Set (eBioscience, 00-5523-00), Fixation/Permeabilization Solution Kit (BD Cytofix/Cytoperm™, 554714), Ultra-LEAF™ Purified anti-mouse IL-4 Antibody (Biolegend, 504135), Ultra-LEAF™ Purified anti-mouse IFN-*γ* Antibody (Biolegend, 505710), Ultra-LEAF™ Purified anti-mouse CD3ε Antibody (Biolegend, 100340), Ultra-LEAF™ Purified anti-mouse CD28 Antibody (Biolegend, 102121), mouse IL-2 (Biolegend, 589102), mouse IL-4 (Biolegend, 574002), mouse IL-6 (Biolegend, 570802), mouse IL-12 (Biolegend, 573002), mouse IL-23 (Biolegend, 574102), mouse TGF-β1 (Biolegend, 781802), mouse IL-1β (PeproTech, 200-01B), PerCP/Cy5.5 anti-mouse CD4 Antibody (PeproTech, 200-01B), FITC anti-mouse IFN-*γ* Antibody (Biolegend, 502506), PE anti-mouse IL-17A Antibody (eBioscience, 12-7178-42), APC anti-mouse IL-4 Antibody (eBioscience, 17-7049-82), APC anti-mouse Foxp3 Antibody (eBioscience, 17-4776-42), mouse CD4^+^ T Cell Isolation Kit (Miltenyibiotec, 130-104-454), Ultra-LEAF™ Purified anti-human CD28 Antibody (Biolegend, 302934), Ultra-LEAF™ Purified anti-human CD3 Antibody (Biolegend, 300438), Ultra-LEAF™ Purified anti-human IL-4 Antibody (Biolegend, 500838), Ultra-LEAF™ Purified anti-human IFN-*γ* Antibody (Biolegend, 506532), human IL-2 (Biolegend, 589102), human IL-4 (Biolegend, 574002), human IL-6 (Biolegend, 570802), human IL-12 (Biolegend, 573002), human IL-23 (Biolegend, 574102), human TGF-β1 (Biolegend, 781802), human IL-1β (PeproTech, 200-01B), PerCP/Cy5.5 anti-human CD4 Antibody (PeproTech, 200-01B), FITC anti-human IFN-*γ* Antibody (Biolegend, 502506), PE anti-human IL-17A Antibody (eBioscience, 12-7178-42), APC anti-human IL-4 Antibody (eBioscience, 17-7049-82), APC anti-human Foxp3 Antibody (eBioscience, 17-4776-42), human CD4^+^ T Cell Isolation Kit (Miltenyibiotec, 130-104-454).

### Patient cohort

Patients with confirmed multiple sclerosis (MS) or neuromyelitis optica spectrum disorder (NMOSD), along with age- and sex-matched healthy controls, were recruited from the Third Hospital of Sun Yat-sen University and the Second Affiliated Hospital of Guizhou University of Traditional Chinese Medicine. Patient characteristics are summarized in Tables S1–S4. All participants provided informed consent. For MS patients, “acute phase” was defined as the presence of new or worsening neurological symptoms consistent with a clinical relapse, within 4 weeks of sample collection, with symptoms lasting at least 24 hours in the absence of fever, infection, or other alternative explanations “Remission phase” was defined as clinical stability for at least 8 weeks prior to sampling. Inclusion criteria included: confirmed diagnosis according to McDonald 2017 criteria (MS) or 2015 IPND criteria (NMOSD), age between 18 and 65 years, and ability to provide informed consent. Exclusion criteria included: pregnancy, active infection, other major systemic inflammatory diseases, receipt of high-dose corticosteroids within 2 weeks before sampling and use of non-steroidal anti-inflammatory drugs within 1 week prior to sample collection. Plasma samples were collected in EDTA-containing tubes and processed within 4 h by centrifugation at 800 × g for 5 minutes. Plasma was stored at –80 °C until analysis. The study was approved by the Institutional Review Board of the Third Hospital of Sun Yat-sen University (Ethical Approval NO. [2020]02-054-01) and the Second Affiliated Hospital of Guizhou University of Traditional Chinese Medicine (Ethical Approval NO. KYW2023012). Clinical variables, including EDSS, disease duration, and medication use, were recorded and, together with age and sex, were included as covariates in multivariable regression models to adjust for potential confounding.

### LC-MS analysis

Plasma from EAE mice was collected 28 days after immunization. Age- and sex-matched naïve mice were used as controls for metabolomic comparisons. Plasma eicosanoids, including PGD_2_, PGE₂, TXB₂, 15d-PGJ₂, and others, were quantified using UHPLC-MS/MS as previously described,^38^ Briefly, samples were sonicated on ice, centrifuged, and subjected to solid-phase extraction. Eluates were analyzed using an I-Class LC system coupled to a QTRAP 5500 (AB Sciex). Chromatographic separation was achieved using a gradient of 0.1% formic acid in water (A) and acetonitrile (B). Ionization was performed in ESI negative mode, and metabolites were quantified using MRM transitions. Data was processed with MultiQuant software.

### ELISA assay

PGD_2_ levels in plasma were measured using a Prostaglandin D2-MOX ELISA Kit (Cayman Chemical) after derivatization with methoxylamine hydrochloride, following the manufacturer’s protocol.

### Real-Time quantitative PCR (RT-QPCR)

Total RNA was extracted and reverse-transcribed using the Transcriptor Universal cDNA Master Kit (Roche). Quantitative PCR was performed using SYBR Green Master Mix (Roche) on a ViiA 7 Real-Time PCR System (Applied Biosystems).

### Mice

C57BL/6 mice were housed under specific-pathogen-free conditions. *Hpgds*^*fl/fl*^ and Cd4-Cre mice were obtained from Cyagen Biosciences. CD4^+^ T cell-specific *Hpgds* knockout mice *Hpgds*^*fl/fl;Cd4-Cre*^ were generated by crossing *Hpgds*^*fl/fl*^ with *Cd4-Cre* mice. For all experiments involving *Hpgds*^*fl/fl;Cd4-Cre*^ mice, the *Hpgds*^*fl/fl*^ mice (Cre-negative) were used as controls. *Pparg*^*-/-*^ mice were kindly provided by Professor Qianjin Lu^39^ (Chinese Academy of Medical Sciences & Peking Union Medical College, Nanjing, China). Genotyping was performed by PCR or qPCR. All animal experiments were approved by the Institutional Animal Care and Use Committee of Macau University of Science and Technology. No animals were excluded from the analysis after randomization.

### EAE Induction and Treatments

EAE mouse model was generated according to previous report.^40^ All mice used for EAE induction experiments were age-matched females between 6 and 8 weeks old at the start of the experimentsue. Briefly, the mice were subcutaneously immunized at three sites in the flank with 100 *μ*L of 400 *μ*g MOG_35-55_ peptide emulsified incomplete Freund’s adjuvant (IFA) supplemented with 4 mg/mL *Mycobacterium tuberculosis* extract H37Ra (BD Difco) on day 0. Pertussis toxin was injected i.p. on days 0 and 2. Mice were scored as follows: 0, no signs; 0.5, mild loss of tail tone; 1.0, complete loss of tail tone; 1.5, mildly impaired righting reflex; 2.0, abnormal gait and/or impaired righting reflex; 2.5, hind limb paresis; 3.0, hind limb paralysis; 3.5, hind limb paralysis with hind body paresis; 4.0, hind and fore limb paralysis; 4.5, moribund; and 5.0, death.^40^ EAE clinical scores were assessed daily by two independent investigators who were blinded to the mouse genotype and treatment group.

Dosage was derived from the FDA-recommended dosage for humans using the Meeh-Rubner formula. Specifically, the HQL-79 dose range was selected based on published studies demonstrating HPGDS inhibition in murine inflammatory models.^27^ Treatment regimens varied by experiment according to the specific mechanistic question being addressed (gain-of-function, loss-of-function, or rescue) and, where applicable, were constrained by phenotype severity and humane endpoint criteria. In detail, for exogenous PGD_2_ administration in wild-type mice: PGD_2_ (500 ng/kg) was administered starting on day 7 post-immunization and continued until day 19. For exogenous PGD_2_ rescue in *Hpgds*^*fl/fl;Cd4-Cre*^ mice: PGD_2_ (1000 ng/kg) was administered starting on day 0 post-immunization and continued throughout the 28-day disease course to compensate for the complete absence of endogenous T cell-derived PGD_2_. For HPGDS inhibition with HQL-79: HQL-79 (5, 10 mg/kg) was administered starting on day 7 post-immunization and continued until day 28. All treatment regimens were continued until the end of the experiment unless otherwise indicated. Animals were monitored daily and euthanized when they reached predefined humane endpoints based on clinical score and overall condition. Mice that failed to develop any clinical signs by day 28 post-immunization were considered non-responders and excluded from the analysis, with the exception of *Hpgds*^*fl/fl;Cd4-Cre*^ mice, in which the absence of clinical signs represents the protective phenotype under investigation. No animals were excluded after the onset of clinical symptoms. All mice included were analyzed following the intention-to-treat principle.

### Histological analysis

Brain and spinal cords were fixed with 4% paraformaldehyde (PFA paraffin-embedded, and stained with hematoxylin and eosin (H&E). Histological analyses were performed on standardized regions including the cerebellum for brain sections, and transverse sections of the lumbar spinal cord. Inflammatory lesions were defined as clusters of ≥10 mononuclear cells and quantified in meninges and parenchyma. Histopathological evaluation was performed by a pathologist blinded to experimental groups.

### Immunohistochemistry

Immunohistochemistry against IL-17A (1:50; Abcam, ab79056) was performed in paraffin-embedded brain and spinal cord sections. Staining with an irrelevant IgG antibody matched to the host species and isotype of the primary antibody also yielded no specific signal. Antigens were visualized following incubation with horseradish peroxidase-conjugated secondary antibodies Anti-IgG (Abcam, ab97064). Analyses were performed on standardized regions including the cerebellum for brain sections, and transverse sections of the lumbar spinal cord. The number of positive immunostained cells in 20 non-overlapping fields (30,000 *μ*m², objective ×20) per sample was analyzed. IHC staining evaluation was performed by a pathologist blinded to the experimental groups.

### Examination of T cell subsets in vivo

Splenocytes and lymphocytes were isolated from EAE mice with indicated treatments. To assess the pre-existing peripheral Th17 cell status ex vivo, cells were stimulated with 100 ng/mL PMA and 1 *μ*g/mL ionomycin for 4–6 h. After that, the cells were stained with PerCP/Cy5.5 anti-mouse CD4, followed by fixed, permeabilized, and stained with PE anti-mouse IL-17A, FITC anti-mouse IFN-*γ*, or APC anti-mouse Foxp3. Finally, the samples were analyzed by flow cytometer, and the acquired data were analyzed using FlowJo software.

### Flow cytometry analysis

For all in vitro T cell differentiation experiments, the following control conditions were included: Control (unstimulated): Naïve CD4^+^ T cells cultured without polarizing cytokines (IL-6, TGF-*β*, IL-23 for Th17 conditions; IL-2, IL-12 for Th1 conditions; IL-2, TGF-*β* for Treg conditions) and without drug treatment. This group represents the baseline frequency of each subset in the absence of differentiation signals. Vehicle: Naïve CD4^+^ T cells cultured under Th17-polarizing conditions with the corresponding solvent control. All compounds were dissolved in DMSO, and the final DMSO concentration was maintained at 0.1% in both vehicle and compound-treated groups. Drug-treated: Naïve CD4^+^ T cells cultured under polarizing conditions and treated with indicated drugs. For analysis the differentiation of T cell subsets, the cells were collected and washed with stain buffer, followed by stained with indicated antibodies and then examined by flow cytometer (BD FACS Aria III). The acquired data were analyzed using FlowJo software.

### Isolation of Naïve CD4^+^ T cells and T cell subsets differentiation

All mice used for in vitro T cell differentiation experiments were age-matched females between 6-8 weeks old at the start of the experiments. Mouse Naïve CD4^+^ T cells were sorted by a biotin selection kit (Miltenyibiotec, 130-104-454) from spleen or LN of C57BL/6 mice. Human T cells were isolated from peripheral blood cells by Ficoll gradient centrifugation (Ficoll-Paque PLUS, GE Healthcare) and then sorted by a biotin selection kit (Miltenyibiotec, 130-096-533). The indicated T cell subsets were polarized according to previous reports.^41^

Briefly, for Th17 cell polarization, naive CD4^+^ T cells were unless otherwise indicated, stimulated with 2 *μ*g/mL anti-CD3, 10 *μ*g/mL anti-CD28 antibodies, 2 ng/ml TGF-*β*1 and IL-23, 20 ng/ml IL-6 for 72 h in RPMI-1640 containing 10% FBS. For Th1 cell polarization, naive CD4^+^ T cells were unless otherwise indicated, stimulated with 2 *μ*g/mL anti-CD3, 10 *μ*g/mL anti-CD28 antibodies, 2 ng/ml IL-2 and 5 ng/ml IL-12 for 72 h in RPMI-1640 containing 10% FBS. For Treg cell polarization, naive CD4^+^ T cells were unless otherwise indicated, stimulated with 2 *μ*g/mL anti-CD3, 10 *μ*g/mL anti-CD28 antibodies, 10 ng/ml IL-2 and 30 ng/ml TGF-*β*1 for 72 h in RPMI-1640 containing 10% FBS.

After stimulation, T cells are cultured in RPMI-1640 medium supplemented with 50 ng/mL PMA, 1 *μ*g/ml ionomycin, 1× Brefeldin A for 4–6 h in 37 °C. The cells were collected by centrifugation at 800 × *g* for 5 min at 4 °C. The samples were analyzed by flow cytometry.

### Binding affinity assay

Recombinant human ROR*γ*t protein was prepared according to the previous method.^42^ Recombinant human PPAR*γ* protein was purchased from Cayman Chemical (61700). The binding kinetics of indicated molecules on PPAR*γ* and ROR*γ*t were evaluated according to previous reports.^43^

### Molecular docking study

Molecular docking studies were performed by the Induced Fit Docking (IFD) module of the Schrodinger software package (Maestro 11.1) referred to previous research. The crystal structures of PPAR*γ* (PDB ID: 5Y2O) were downloaded from protein data bank (www.rcsb.org). The PPAR*γ* protein was prepared in the Protein Preparation Wizard by repairing the missing residues, adding hydrogen, removing water and minimized the structure. The 3D structure of PGD_2_ were constructed in Maestro and further optimized with the LigPrep panel. The binding site of protein was set at a 30 Å × 30 Å × 30 Å box in the center of initial compound. After the initial Glide docking was applied, the ligand and the side chains within 6.0 Å in each initial poses were taken into prime refinement for optimization. Next, Extra-precision (XP) docking was employed to redock compounds into the binding site. The conformer with the best score was analyzed and visualized in PYMOL.^44,45^

### CHIP-assay

Splenocytes were isolated from C57BL/6 mice. The anti-PPAR*γ* antibody (Cell Signaling Technology: Antibodies, 2435) was used for ChIP assays. Briefly, 1 million cells were washed with cold phosphate-buffered saline (PBS) and lysed in buffer containing protease inhibitor cocktail. Mononucleosomes were generated in digestion buffer containing 6 U of micrococcal nuclease, and diluted supernatants were immunoprecipitated with the indicated antibody. A total of 10% of the diluted supernatants were kept as input. Immunoprecipitated DNA and input DNA were assessed by real-time PCR.^46^ The primer sequences are as follows:

Fabp4 promoter:

Forward: 5’-CAGGGTCTGGGAGAAGGTTA-3’

Reverse: 5’-GCTGGTTGAGGTTCTGGTGT-3’

LPL promoter:

Forward: 5’-GCTGGAGGTGGAAGAGGAAT-3’

Reverse: 5’-CAGGGTCAGGCTTCCTCTTC-3’

Perilipin promoter:

Forward: 5’-CAGCTGGGACTTGGTGTCTT-3’

Reverse: 5’-TCCCCACAGTTTCCCTCTTC-3

### Preparation of recombinant PPAR*γ* protein

The gene of full length PPAR*γ* protein and its mutants (NCBI Gene ID 5468) were constructed and cloned into pET28. In brief, the mutations in PPAR*γ*, including histidine 266 (H266), arginine 288 (R288), serine 289 (S289), serine 342 (S342), and glutamate 343 (E343) mutated to alanine (A) were constructed to obtained H266A, R288A, S289A, S342A, and E343A according to the docking result. BL21 (DE3) competent *Escherichia coli* cells then were transfected with indicated mutant plasmid. The cells were grown in Luria-Bertani liquid and induced with isopropyl β-d-1- thiogalactopyranoside (IPTG, 0.5 mM) overnight at 16 °C. Mutants PPAR*γ* protein were purified from bacterial lysates using a HisTrap FF column (GE Healthcare, WI, USA).

### Promoter luciferase assay

*IL17A* LUCPorter and *PPARG* LUCPorter Stable Reporter cells were plated (2 × 10^4^ cells/well) in 96-well plates. The cells were incubated with different concentrations of PGD_2_ for 16 h. The luciferase activity of *IL17A* or *PPARG* were assessed according to previous reports.^41^

### Statistics

Data are presented as mean ± Standard Error of the Mean (SEM). Normality was assessed using the Shapiro-Wilk test. Homogeneity of variances was evaluated using an F test for two-group comparisons and the Brown-Forsythe test for comparisons among more than two groups.

Clinical EAE scores over time were analyzed using two-way repeated-measures ANOVA with Šídák’s post hoc test for multiple comparisons. For comparisons between two independent groups, an unpaired two-tailed Student’s *t* test was used when assumptions were met; when variances were unequal, Welch’s correction was applied. For comparisons among more than two groups, data was analyzed by ordinary one-way ANOVA. When the overall ANOVA was significant, post hoc comparisons were performed using Dunnett’s multiple comparisons test; when variances were unequal, Welch’s one-way ANOVA was performed with an appropriate post hoc test for unequal variances. When assumptions were not met, non-parametric tests were used (Mann–Whitney U test for two-group comparisons; Kruskal-Wallis test with Dunn’s multiple-comparisons test for more than two-group comparisons). Correlations between clinical parameters and PGD_2_ concentrations were assessed using Spearman correlation analysis, and partial correlation analysis was performed after adjusting for potential confounders. A two-sided *p* value < 0.05 was considered statistically significant. Analyses were performed in GraphPad Prism 10 (GraphPad Software) or R software version 4.3.3 (R Foundation for Statistical Computing, Vienna, Austria).

## Supplementary information

Supplementary Materials

Supporting data values

Gating strategies

Video S1A Clinical symptoms of *Hpgds*^*fl/fl, CD4-Cre*^ mice recorded on day 28 post-immunization

Video S1B Clinical symptoms of *Hpgds*^*fl/fl, CD4-Cre*^ mice recorded on day 28 post-immunization

Video S2A Clinical symptoms of *Hpgds*^*fl/fl, CD4-Cre*^ mice treated with 1,000 ng/kg PGD_2_ recorded on day 28 post-immunization

Video S2B Clinical symptoms of *Hpgds*^*fl/fl, CD4-Cre*^ mice treated without 1,000 ng/kg PGD_2_ recorded on day 28 post-immunization

## Data Availability

All the data supporting the results of this study are included in the main text and its Supplementary Information. The clinical data of patients used in this study are available upon reasonable request, and all patient information will be anonymized to protect confidentiality. Requests for access to the clinical data should be addressed to L.T. (tli@must.edu.mo).
