# Supplementary material for: Targeting the HPGDS-PGD2 axis to treat Th17 cell-mediated autoimmunity in multiple sclerosis
Source: Signal Transduct Target Ther. 2026 Oct 1;11:420. doi: 10.1038/s41392-026-02937-5 (PMC13627710; doi:10.1038/s41392-026-02937-5)
Supplement: Supplementary file 1 [file 41392_2026_2937_MOESM1_ESM.docx]

Supplementary Materials for

Targeting the HPGDS-PGD_2_ axis to treat Th17 cell-mediated autoimmunity in multiple sclerosis

Yuxin Zhuang, Kawai Lei, Yan Li, Wende Deng, Jianmin Yue, Jianbo Wan, Qibiao Wu, Qianjin Lu, Yun Tang, John E.J. Rasko, Juan Liu, Olivia Monteiro, Xiqing Bian, Wei Qiu, Ting Li.

Correspondence to: tli@must.edu.mo (Ting Li), qiuwei@mail.sysu.edu.cn (Wei Qiu).

**This PDF file includes:**

Figures S1 to S19

Tables S1-S5

Captions for Videos S1 to S2

**Other Supplementary Materials for this manuscript include the following:**

Videos S1 to S2

Figure. S1. Association between plasma PGD_2_ levels and diagnosis (MS vs. NMOSD) -multivariable logistic regression models. Model 1 was adjusted for sex and age. Model 2 was additionally adjusted for medication use (treatment-naive, glucocorticoids, and conventional immunosuppressants). Model 3 was further adjusted for current clinical status (acute vs remission), EDSS score, and disease duration (months), in addition to the variables included in Model 1. Plasma PGD_2_ was modeled per 100 pg/mL increase. Abbreviations: OR, odds ratio; CI, confidence interval.

Figure. S2. Sensitivity analysis of association between plasma PGD_2_ and disease classification (MS versus NMOSD). Panel A: included patients with MS and NMOSD in remission only (n = 62). Model 1 was adjusted for sex and age; Model 2 was additionally adjusted for medication use (treatment-naive, conventional immunosuppressants), and EDSS. Panel B: included patients with RRMS and NMOSD only (n = 93). Model 1 was adjusted for sex and age; Model 2 was additionally adjusted for current clinical status (acute vs remission), medication use (glucocorticoids, conventional immunosuppressants), and EDSS. Panel C: included patients not treated with glucocorticoids (n = 69). Model 1 was adjusted for sex and age; Model 2 was additionally adjusted for current clinical status (acute vs remission), treat with conventional immunosuppressants, and EDSS. Plasma PGD_2_ was modeled per 100 pg/mL increase. Abbreviations: OR, odds ratio; CI, confidence interval; EDSS, Expanded Disability Status Scale.

Figure. S3. Association between Plasma PGD_2_ levels and Expanded Disability Status Scale (EDSS). (a) Spearman correlation analysis showing a positive association between PGD_2_ and EDSS. (b) Partial correlation analysis adjusting or age and sex and medication use (treatment-naive, glucocorticoids, and conventional immunosuppressants).

Figure. S4. Flow cytometric analysis of Th17 cell in peripheral CD4^+^ T cells from MS patients.

Figure. S5. PGD_2_ does not promote Th1 or Treg cell differentiation in MS patients. Naive CD4^+^ T cells from MS patients were cultured under Th1 or Treg cells conditions with increasing concentrations of PGD_2_ for 3 days. Th1 and Treg cells were analyzed by flow cytometry (n=3). Data are represented as mean ± SEM.

Figure. S6. PGD_2_ exacerbates EAE severity in a dose- and timing-dependent manner. (a-b) Body weight and EAE clinical scores in C57BL/6 mice treated with 500 or 1,000 ng/kg PGD_2_ on day 7 post-immunization (n=6). (c-d) Body weight and EAE clinical scores in mice treated with 500 ng/kg PGD_2_ on day 0 or day 7 (n=6). Data are represented as mean ± SEM.

Figure. S7. Validation of IL-17A IHC specificity using an isotype control. (a) isotype control IgG staining of brain section. (b) isotype control IgG staining of spinal cord section. 20× objective, Scale bars, 200 *μ*m.

Figure. S8. PGD_2_ enhances Th17 but not Th1 or Treg cell differentiation in EAE mice. Proportions of Th1 and Treg cells in CD4^+^ T cells isolated from spleen (a) and LN (b) of EAE mice treated with or without PGD_2_ (500 ng/kg) on day 19 post-immunization (n=6). Data are represented as mean ± SEM.

Figure. S9. PGD_2_ does not promote Th1 or Treg cell differentiation *in vitro*. Naive CD4^+^ T cells were cultured under Th1 or Treg conditions with indicated concentrations of PGD_2_. Th1 and Treg frequencies were determined by flow cytometry (n=3). Data are represented as mean ± SEM.

Figure. S10. The effect of LPGDS inhibitor AT-56 on Th17 cell differentiation *in vitro*. Naive CD4^+^ T cells were cultured under Th17 cell conditions with increasing concentrations of AT-56. Th17 cells were analyzed by flow cytometry (n=3). Data are represented as mean ± SEM.

Figure. S11. Time-dependent induction of Th17 cell differentiation and *Il17a* expression. Naive CD4^+^ T cells cultured under Th17 cell-polarizing conditions were analyzed for Th17 cell frequency (a) and *Il17a* mRNA expression (b) over time (n=3). Data are represented as mean ± SEM. *****p* < 0.0001.

Figure. S12. HPGDS Expression in CD4^+^ T cells under different polarizing conditions. Naive CD4^+^ T cells cultured under Th1, Th17, or Treg cells conditions were stained for HPGDS protein expression by flow cytometry.

Figure. S13. CD4^+^ T cell-specific deletion of *Hpgds* protects against EAE without affecting Th1 or Treg differentiation. Frequencies of Th1 and Treg cells in spleen (a) and LN (b) of *Hpgds^fl/fl^* and *Hpgds^fl/fl;Cd4-Cre^* mice at day 28 post-immunization (n=6). Data are represented as mean ± SEM.

Figure. S14. PGD_2_ restores EAE susceptibility in *Hpgds^fl/fl;Cd-Cre^* mice without altering Th1 or Treg differentiation. Frequencies of Th1 and Treg cells in spleen (a) and LN (b) of *Hpgds^fl/fl;Cd4-Cre^* mice intraperitoneally injected with PGD_2_ (1,000 ng/kg) (n=6). Data are represented as mean ± SEM.

Figure. S15. PGD_2_ does not interact with RORγt or directly induce IL-17A transcription. (a) BLI assay showing no detectable interaction between PGD_2_ and RORγt. (b) PGD_2_ had no effect on *IL-17A* promoter luciferase activity (n=3). Data are represented as mean ± SEM.

Figure. S16. Th17 cell differentiation is independent of DP1 or CRTH2 Signaling. Flow cytometric analysis of Th17 cell cultured with the DP1 inhibitor Asapiprant (a) or CRTH2 inhibitor Ramatroban (b) (n=3). Data are represented as mean ± SEM.

Figure. S17. BLI binding curves of PGD_2_ to PPAR*γ* with point mutations.

Figure. S18. Direct PGD_2_-HPGDS interaction measured by BLI. Biolayer interferometry binding curves showing concentration-dependent binding of HQL-79 to recombinant human HPGDS protein.

Figure. S19. HQL-79 does not affect Th1 or Treg differentiation in EAE mice. (a-b) Th1 and Treg frequencies in spleen (a) and LN (b) of EAE mice treated with or without HQL-79 on day 28 post-immunization (n=5-6). Data are represented as mean ± SEM.

Table S1.

Clinical information of the MS patients cohort.

Table S2.

Clinical information of the NMOSD patients cohort.

Table S3.

Clinical information of the healthy control cohort.

Table S4.

The clinical characteristics of the investigated population.

Table S5.

Clinical information of MS patients donating peripheral blood for *ex vivo* CD4^+^ T cell experiments.

Video S1.

Clinical symptoms of *Hpgds^fl/fl^* (a) and *Hpgds^fl/fl;Cd4-Cre^* (b) mice recorded on day 28 post-immunization.

Video S2.

Clinical symptoms of *Hpgds^fl/fl;Cd4-Cre^* mice treated with (a) or without (b) 1,000 ng/kg PGD_2_ recorded on day 28 post-immunization.
